# Do Older and Younger Adults Prefer the Positive or Avoid the Negative?

**DOI:** 10.3390/brainsci12030393

**Published:** 2022-03-15

**Authors:** Beth Fairfield, Caterina Padulo, Alessandro Bortolotti, Bernardo Perfetti, Nicola Mammarella, Michela Balsamo

**Affiliations:** 1 Department of Humanistic Studies, University Federico II of Naples, 80138 Naples, Italy; beth.fairfield@unina.it; 2 Department of Psychological, Heath and Territorial Sciences, University of Chieti, 66013 Chieti, Italy; perfetti@inwind.it (B.P.); nicola.mammarella@unich.it (N.M.); michela.balsamo@libero.it (M.B.); 3 Department of Neuroscience, Imaging and Clinical Sciences, University of Chieti, 66013 Chieti, Italy; alessandro.bortolotti@unich.it

**Keywords:** emotion-cognition interactions, implicit processing, affective information, aging, positivity effect

## Abstract

Affective information is processed in different ways across one’s lifespan. Explanations for this pattern of performance are multiple and range from top-down motivational shifts and cognitive control to faster bottom-up and implicit processes. In this study, we aimed to investigate implicit affective information processing and positivity effects by examining performance in a modified version of the dot-probe task across three image-pair conditions (positive/neutral; negative/neutral; and positive/negative). We examined data from 50 older adults and 50 younger adults. The results showed that affective information processing varies with age and valence and that age effects in affective processing may occur early during information processing. Positivity biases emerge in both younger and older adults. However, while younger adults seem to prioritize positive information independently of context, older adults showed this prioritization only when presented in an emotional (i.e., negative) context. Moreover, older adults showed a tendency to avoid negative information whereas younger adults showed a general bias for affective content modulated by image-pair context.

## 1. Introduction

Positivity effects, a preference for positive stimuli compared to negative and neutral [1,2], have been repeatedly associated with successful aging [3,4] and have been found within different cognitive functions including memory [5,6], decision making [7], and attention [5].

Some explanations assume that it is generated by top-down motivational shifts towards emotional content, since cognition in older adults is generally more motivated toward emotion processing and regulation than younger adults (the socioemotional selectivity theory, SST) [8]. In addition, a cognitive-control-based explanation posits that older adults consciously allocate cognitive resources to prioritize positive information and assign positive valence to an event via conscious reflective processes [9,10]. Consequently, positivity seems to be a function of available cognitive resources and selective allocation of these resources towards prioritized emotional goals [9]. Other explanations, instead, posit that positivity may also result from age-related cognitive [11] or neurological declines [12]. Indeed, whatever the top-down motivational explanation be, older adults generally show reduced speed of processing, difficulties in inhibition, and are more subject to interference. These declines may modulate top-down goal-driven and experience-based processes. From this point of view, older adults’ preference for positive stimuli may occur early in the cascade of cognitive processes as a way of simplifying information processing. For example, Mather and Knight [9] found that older adults show a preference for positive images if they are not asked to simultaneously carry out a cognitively demanding task. These researchers posited that older adults may revert to a sort of “habitual” emotion information processing strategy to simplify information processing by prioritizing positive information and eventually de-prioritizing negative information unless cognitive resources are required [13]. Thus, there may possibly exist multiple and different pathways, related to bottom-up or top-down processes, leading to positivity [11,14,15].

Moreover, preferences for positive information may also be the result of two different information processing strategies. On the one hand, positivity biases result from older adults’ prioritization of positive over negative information. On the other hand, positivity biases could also be the result of avoiding negative stimuli (that is, the preference for positive information emerges due to a de-prioritization of negative information).

Many studies in the literature have adopted the dot-probe task [16,17], to investigate bottom-up versus top-down information processing. In the dot probe task participants are instructed to stare at a fixation cross in the center of the screen. Then, two visual stimuli, one neutral and one affective, appear randomly on the screen (one left and one right). Following a fixed interval, a dot is presented in the position of one of the previous stimuli and participants are instructed to indicate the position of the dot as quickly as possible Results show faster response times when the dot is in the position of the affective stimulus [18]. In particular, studies investigating the differential effect of affective (positive or negative) and neutral stimuli on responding to target probes in older adults (for a review see [19]) report slower response times when target probes replace negative stimuli compared to neutral ones [5,20]. On the contrary, younger adults show no variations in response times. Authors interpret older adults’ slower performance for probes replacing negative stimuli as a tendency to avoid negative instead of a preference for positive stimuli. Interestingly, this pattern emerges with longer cue presentation compared to shorter presentation, suggesting that the age-related bias in the negative condition is likely mediated by active control mechanisms [14,19,21]. However, these studies evaluated only the competition between an affective image (positive or negative) and a neutral one. Only a few studies have evaluated the effect of direct competition between a negative and a positive stimulus in the dot-probe task [6]. These researchers found a negative bias in the negative-positive pair but only in younger participants. Moreover, as in previously mentioned studies [19,21], this pattern emerges only when the cue is presented for a sufficiently long interval, suggesting again a mediation of active top-down control mechanisms during affective information processing. Few studies have investigated short time intervals [20,22], and none of these has reported significant effects of implicit bottom-up affective information processing. None of these studies directly compared competition between positive and negative stimuli, a manipulation that would allow clarifying bottom-up influences during affective information processing.

Here, our aims are threefold. First, we aimed to further explore the differential effect of affective and neutral stimuli on responding to probes by directly comparing positive and negative information as well as affective-neutral pairs. Second, we addressed a limitation of previous investigations regarding the duration of emotional stimuli presentation. Here, we limited presentation intervals to 100 ms.

Third, we explored the effects of complex images [23], rather than faces, since pictures are more potent than facial expressions at inducing changes in the subjective evaluations of emotional valence and arousal [24].

According to the dynamic integration theory [11], we expect to find implicit emotion processing differences early in the cascade of information processing. If older adults’ preference for positive information is the result of processing strategies aimed at simplifying information processing by prioritizing positive information, we expect older adults to show better performance with positive compared to neutral and negative information independently of context. Otherwise, if this preference is the result of de-prioritization of negative information, we expect better performance with positive compared to negative information especially in an emotional context and, perhaps, with neutral compared to negative information.

## 2. Materials and Methods

### 2.1. Participants

Fifty-one undergraduates from the University of Chieti (41 females (82%), 19–27 age range, 20 median age) and 52 older adults recruited from the local community through a snowball sampling procedure starting from undergraduates and local craftsmen (35 females (70%), 63–88 age range, 70 median age) participated in the study. Average years of education were 15.20 (SD = 1.1) and 6.94 (SD = 2.8) for the younger and older adults, respectively. All participants were right-handed, native Italian speakers, had normal or corrected to normal vision and with no reports of psychiatric or neurological disorders (nor use of psychiatric drugs). Inclusion criteria included a Mini Mental State Examination score > 24 for older adults. Participants did not receive monetary reimbursement for their participation. All participants signed informed consent forms approved by the Institutional review board.

Neuropsychological and demographic data are reported in Table 1. First, general cognition was assessed with the Culture-Fair Intelligence Test [25] in the younger group and with the Mini Mental State Exam (MMSE) [26] and the Raven Colored Progressive Matrices [27] in the older adults. Responses were collected orally by the experimenter. Then, all participants completed an online computer collected response version of the “State Trait Inventory of Cognitive and Somatic Anxiety” (STICSA) [28], to assess cognitive, somatic and anxiety symptoms and the “Teate Depression Inventory” (TDI) [29], to assess major depressive disorder (DSM-V) [30,31]. We did not find any reliable differences in anxiety (state and trait) or depression. We excluded two older adults from the analyses in line with exclusion criteria (MMSE < 24) and one younger adult for incomplete data.

### 2.2. Materials

We selected 180 images from the International Affective Picture System (IAPS) [23] according to normative data for valence, arousal and dominance in younger and older adults [32,33,34], and discrete categories such as happiness, anger, and fear [35,36]. Picture inclusion criteria were: valence ratings for unpleasant stimuli <25th percentile on both datasets and >75th percentile on the “anger and fear” dimension; valence ratings for the pleasant images >75th percentile on both datasets and >75th percentile on the “happiness” dimension; neutral stimuli within the range of mean ± 1/3 SD in both datasets.

### 2.3. Experimental Task and Procedures

**Figure 1:**
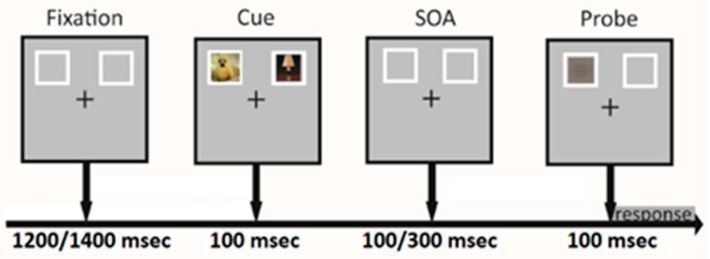
Dot-probe task. The figure displays the sequence and timing of events within a trial. Two examples of cue are presented along with a target probe.

Each trial began with a fixation cross at the center of the screen with two rectangular frames, one on the left and one on the right of the computer monitor. Immediately after, two images were presented simultaneously inside the two frames for 100 ms followed by a delay lasting randomly for 100, 150, 200, 250, 300 ms. Next, a single target appeared inside either the left or right rectangular frame for 100 ms (Figure 1). Targets were horizontal or vertical bars. Participants received instructions before beginning each block of trials. For half of the blocks, they were instructed to press the spacebar as quickly as possible for targets with a horizontal orientation. For the other half, they were instructed to press the spacebar as quickly as possible for targets with a vertical orientation. We asked participants to fix on the central cross for the whole experimental task. They were explicitly told that the images preceding the target were not informative of its location. 

All participants completed a practice session of 20 trials. We constructed a total of 720 trials, divided in 6 blocks, for the experimental task. Specifically, in three blocks of trials participants responded to horizontal targets while they responded to vertical targets in the remaining three. Horizontal and vertical blocks were alternated and half of the participants began with a horizontal block and half with a vertical block. We manipulated affective processing by presenting three different types of image pairs: negative/neutral, positive/neutral, and negative/positive. The presentation of image pairs was balanced, randomly within blocks and had an equal number of occurrences (240 trials each). Each image appeared four times within the entire task. The experimental session lasted about 30 min. All participants were tested in person in the university laboratory by student research assistants.

## 3. Results

Statistical analyses were performed using StatSoft STATISTICA 8.0.550. For each participant, we computed accuracy rates, calculated as the difference between correct responses to targets and errors (range 0–1; Table 2), and reaction times (RTs) on targets for each condition. The different conditions were: target in the same position of a negative image presented with a neutral one (NEGnu), target in the same position of a negative image presented with a positive one (NEGpo), target in the same position of a neutral image presented with a negative one (NEUne), and target in the same position of a neutral image presented with a positive one (NEUpo) target in the same position of a positive image presented with a neutral one (POSnu), and target in the same position of a positive image presented with a negative one (POSne). We entered mean accuracy and RTs into a series of General linear models (GLM). When a significant effect was found, a Tukey’s correction for multiple comparisons was applied to post hoc comparisons.

### 3.1. Accuracy

We carried out an initial general analysis to examine how affective and neutral pictures in a neutral context (positive-neutral, negative-neutral, neutral-positive, and neutral-negative) affect performance. In another analysis, we aimed to verify how different contexts affected performance. That is, we examined performance for negative and positive pictures paired with both neutral and affective contexts (positive and negative, respectively). We carried out an initial GLM with condition (four levels: NEGnu, POSnu, NEUne, NEUpo) as within-subject factor and group (two levels: younger vs older) as between-subjects factor. Analyses revealed a main effect of group since younger adults performed better than older adults (F(1,100) = 21.603, *p* < 0.001, η^2^ = 0.178, power to detect the effect was 0.996). We also found a main effect of condition (F(3,300) = 4.8826, *p* = 0.002, η^2^ = 0.05, power to detect the effect was 0.906). Post hoc analyses revealed that all participants performed better with targets in the POSnu condition compared to targets in the neutral (NEUpo: *p* = 0.004, NEUne: *p* = 0.05) and NEGnu conditions (*p* < 0.009). The two-way interaction between condition and group was also significant (F(3,300) = 3.5051, *p* = 0.02, η^2^ = 0.03, power to detect the effect was 0.778). Post hoc comparisons revealed that only younger adults performed better on targets in the POSnu condition compared to targets in the neutral condition (NEUpo: *p* < 0.001, NEUne: *p* = 0.03). No other significant comparisons were found (all *p* > 0.174). 

**Figure 2:**
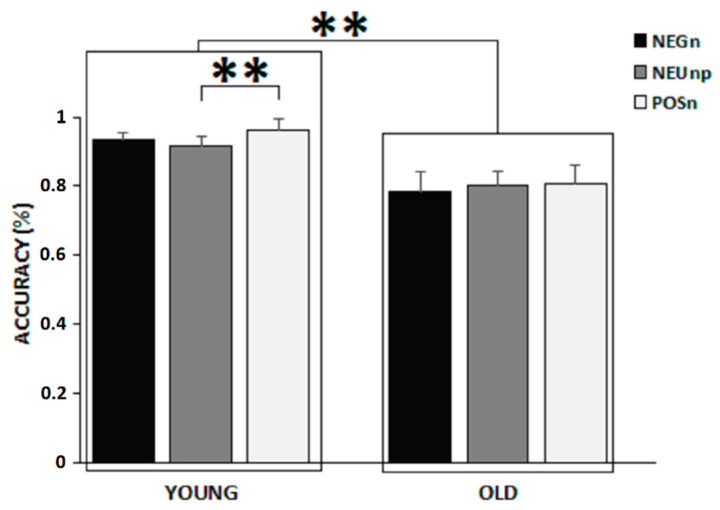
Interaction: condition × group. Error bars represent standard errors. ** *p* < 0.01.

Given that there were no differences between accuracy on target associated to neutral image pairs in affective contexts (i.e., NEUne and NEUpo) for both young and older adults (all *p* > 0.951), we collapsed mean accuracies on targets associated to neutral image pairs to create a single neutral target image and affective context (NEUnp) and computed a second GLM with condition (three levels: NEGnu, POSnu, NEUnp) as within-subject factor and group (two levels: young vs old) as between-subjects factor. Analyses revealed again a main effect of group as young adults’ mean accuracy was greater than older adults (F(1,100) = 23.279, *p* < 0.001, η^2^ = 0.189, power to detect the effect was 0.998), and a main effect of condition (F(2,200) = 7.3327, *p* < 0.001, η^2^ = 0.07, power to detect the effect was 0.936) with greater accuracy for targets in the POSnu condition compared to targets in both NEUnp (*p* = 0.004) and NEGnu (*p* < 0.002) conditions. There was also a significant interaction condition × group (F(2,200) = 3.9848, *p* = 0.02, η^2^ = 0.04, power to detect the effect was 0.709; Figure 2). Post hoc comparisons revealed that only young adults performed better on targets in the POSnu condition compared to targets in the NEUnp condition (*p* < 0.001).

Finally, to better understand whether results were guided by a facilitation for positive information or an avoidance of negative information or both, we conducted a final analysis on affective target images and their contextual pair image (NEGpo, NEGnu, POSne, POSnu) alone. We excluded neutral target images with affective context pairs from the analysis (NEUne and NEUpo). Specifically, we carried out a third GLM with target Image (two levels: NEG vs POS) and context pair type (two levels: neutral vs emotional) as within-subject factors and group (two levels: young vs old) as between-subjects factor. Analyses revealed a main effect of group since younger adults performed better than older adults (F(1,100) = 23.820, *p* < 0.001, η^2^ = 0.192, power to detect the effect was 0.998), a main effect of image condition (F(1,100) = 321.43, *p* < 0.001, η^2^ = 0.76, power to detect the effect was 1.0) with greater accuracy for targets associated with positive images compared to negative ones, and a main effect of pair type (F(1,100) = 29.647, *p* < 0.001, η^2^ = 0.23, power to detect the effect was 1.0) with greater accuracy for targets associated with neutral pair type compared to targets associated to emotional pair type. Moreover, mean accuracy on targets associated with positive images was greater than accuracy on targets associated with negative images in both neutral (*p* = 0.03) and emotional (*p* < 0.001) pair types.

We also found a significant two-way interaction between pair type and group (F(1,100) = 6.7047, *p* = 0.01, η^2^ = 0.06, power to detect the effect was 0.727). Post hoc comparisons revealed that only younger adults performed better on targets associated with neutral pair type compared to targets associated with emotional pair type (*p* < 0.001).

**Figure 3:**
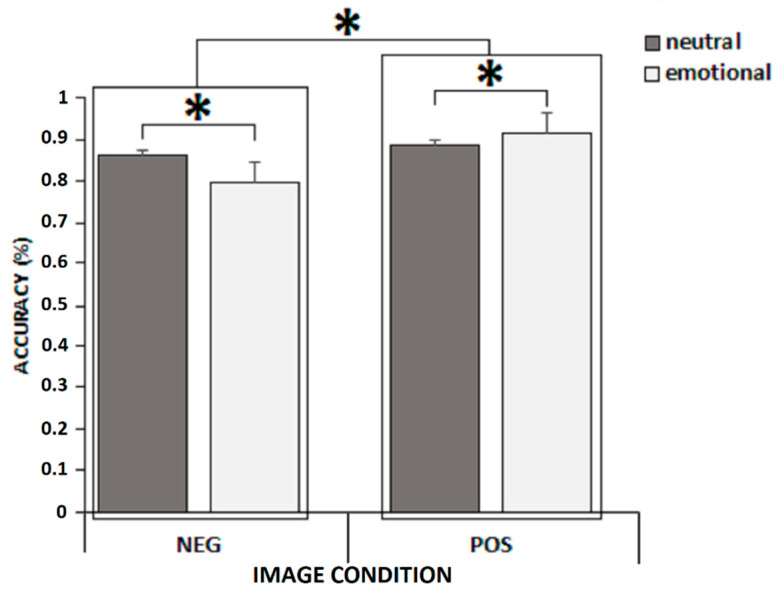
Interaction between image condition and pair type. Error bars represent standard errors. * *p* < 0.05.

The two-way interaction between image condition and pair type was also significant (F(1,100) = 51.345, *p* < 0.001, η^2^ = 0.34, power to detect the effect was 1.0 (Figure 3)). Post hoc comparisons revealed that while accuracy on targets associated with positive images was greater when in an emotional pair (i.e., paired with a negative image) compared to a neutral one (i.e., paired with a neutral image) (*p* = 0.02), mean accuracy on targets associated with negative images was greater when paired with neutral compared to an emotional (i.e., positive) one (*p* < 0.001). 

**Figure 4:**
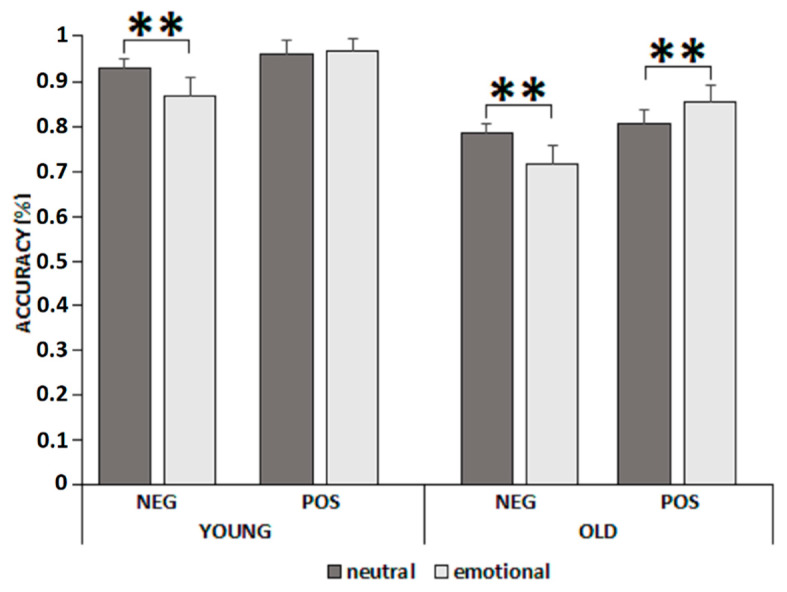
Interaction image condition × pair type × group. Error bars represent standard errors. ** *p* < 0.01.

Although the three-way interaction image condition × pair type × group showed only a trend (*p* = 0.08; Figure 4), planned comparisons showed that younger adults showed lower accuracy on targets associated with negative images in the emotional pair type than in the neutral one (*p* < 0.001) and no differences on targets associated with positive images in the neutral or emotional pair type (*p* = 0.999). Differently, older adults showed lower accuracy on targets associated with negative images in the emotional pair type than in the neutral one (*p* < 0.001) and greater accuracy on targets associated with positive images in the emotional pair type than in the neutral one (*p* = 0.007).

### 3.2. RTs

Analyses on RTs only showed a main effect of group (F(1,100) = 15.097, *p* < 0.001, η^2^ = 0.13, power to detect the effect was 0.970) since younger adults were faster than older adults across all conditions. All other analyses conducted on RTs were not significant (all *p* > 0.05).

## 4. Discussion

Affective information is processed in different manners across the life span. Indeed, compared to younger adults, older adults seem to remember positive information better than negative and neutral information [37,38], but exactly when affective information, and positive information in particular, influences the cascade of cognitive mechanisms underlying information processing is still under debate. In addition, positivity biases may also be the result of different processing strategies used during information processing. That is, positivity biases may emerge due to the fac that positive information is prioritized with respect to the negative information. Differently, positivity biases may also emerge since negative information is deprioritized compared to positive information. 

The present study aimed to investigate affective information elaboration and positivity biases within the context of early implicit processing by examining performance in a modified version of the dot-probe task. Specifically, our study aimed to clarify whether older adults’ preference for positive information was the result of prioritizing positive information and/or de-prioritizing negative information. 

In line with most previous studies, we found that younger adults performed better than older adults [39,40], both in terms of accuracy and mean reaction times. In addition, affective information was processed in different ways across the life span according to the valence of the paired image. 

In a neutral context (paired image neutral), younger adults showed positivity biases and identified targets better when these appeared in a position previously occupied by a positive image compared to a negative one. Instead, older adults were not affected by the valence of the preceding image. Although this finding is unexpected, it should be noted that the literature in this domain is quite conflicting. In fact, while several studies found positivity biases in older adults and negativity biases in younger adults [4,37], other studies found conflicting results, as for example, no age difference in the threat-detection advantage [9], better performance of both younger and older adults with positive and negative images than neutral ones [41], and no valence effect in older adults with comparable performance with positive, negative and neutral stimuli as well [37]. Obviously, at least partially, the different methodologies, tasks and stimuli used could account for the inconsistent outcomes.

More interestingly, when we directly compare implicit affective information processing (i.e., positive vs negative) according to the paired image (affective or neutral), younger and older adults show important differences. The specific (emotional or neutral) context did not affect performance for positive images in younger adults. Instead, older adults performed better when the positive image was presented in a negative context compared to a neutral one. Differently, when targets appear in a position previously occupied by a negative image, both younger and older adults performed better when the context was neutral. 

Since images were presented for only 100 ms, these results suggest that the preference for positive images may occur very early in the cognitive cascade. More importantly, we found that younger and older adults process affective information differently when positive and negative information are presented simultaneously. Younger adults seem to prioritize positive information independently of the paired information, suggesting a general preference for positive information, even early during information processing. Instead, older adults showed context dependent advantages for positive images suggesting that positivity biases in older adults may be linked to bottom-up processing that, on the one hand, seems to prioritize positive information while on the other, seems to avoid negative information. That is, prioritization since older adults perform poorer when the paired context is positive, and avoidance since older adults perform better when the paired context is negative. That is, when older adults are presented with affective image pairs (positive-negative or negative-positive), they perform poorer when the target corresponds to a position that was previously occupied by a negative image, suggesting that older adults are “distracted” by the paired positive context. On the contrary, they perform better when the target corresponds to a position that was previously occupied by a positive image, suggesting a processing preference that leads them to “avoid” the negative context. Importantly, such an outcome occurs very early in the cognitive cascade, suggesting that it does not depend on cognitive-control-based mechanisms. Thus, in line with Kennedy et al. [13], our results suggest that older adults may revert to a sort of “habitual” emotion information processing strategy aimed at simplifying information processing by both prioritizing positive information and de-prioritizing negative information. This bias toward specific emotional content may occur at very low-level processes. We also speculate that avoiding negative information may be a low-level mechanism linked to age-related changes in motivational saliency. Although this outcome is in contradiction to Gronchi et al. results [14], it must be considered that emotional information generally rapidly captures attention and often evokes automatic response tendencies. Positive information motivates approach, while negative information encourages avoidance [42,43]. Thus, it is possible that both bottom-up and top-down processes embrace such responses. Reaction times seem to support this explanation. Although unexpected, we found no differences between younger and older adults across conditions. It may be that since target presentation was limited to 100 ms both groups may have used more bottom-up processing strategies that may mask eventual age-related differences. In addition, the advantage that younger adults typically show in face processing, may not extend to complex images that include more detailed information to process. In addition, the lack of timing differences between targets associated with positive or negative images independently of context, suggest that both prioritizing positive information and de-prioritizing negative information might be characteristic of faster implicit emotional processing. Here we found that affective information processing not only differs across the adult lifespan but that differential processing can be observed even very early. At 100 ms younger and older adults already show processing differences for affective images. Considering this and the behavioral results obtained in this study, neurophysiological research investigating the possibility that different neural mechanisms are responsible for age-related positivity biases need to be carried out. These results may be crucial for designing and implementing prevention and promotion protocols in the general population and especially for healthy older adults. In particular, protocols may be more effective if positive and negative information is presented in competition rather than focusing on isolated positive or negative manipulations (and especially in more demanding cognitive tasks such as memory).

Nonetheless, our study is not without limitations. The images we used widely vary on perceptual features above and beyond emotional content (i.e., valence, arousal, dominance, and category). Although we did control for age differences in valence, arousal and dominance ratings, future studies are needed to evaluate perceptual details such as brightness, contrast and/or luminance to further clarify possible alternative explanations of the complex pattern of results reported here. Finally, even though we did not find reliable differences between younger and older adults in the anxiety and depression dimensions, we cannot exclude possible influences of individual differences on sociodemographic variables such as educational level and/or positive and negative affective state. Also, younger adults showed almost ceiling effects for positive images. Since the task was designed for older adults as well, it may be that it was too simple for the younger adults masking effects that otherwise may emerge. Future studies should address this issue by adopting more demanding tasks.

## Figures and Tables

**Table 1:** Participants’ demographic characteristics.

	Groups			
	Younger—*n* = 50		Older—*n* = 50	
	Mean	SD	Mean	SD
Gender (M/F)	9/41		15/35	
Age (years)	21.2	1.9	70.3	6.2
Years Education *	15.2	1.1	6.9	2.8
Culture Fair	24.0	4.1	-	-
Raven	-	-	24.4	3.3
MMSE	-	-	27.4	1.2
STICSA Trait	34.6	7.4	33.8	6.8
STICSA State	30.5	6.2	32.5	6.6
TDI	44.5	8.9	48.6	9.2

* = *p* < 0.01.

**Table 2:** Mean accuracy rates for each condition and for younger and older adults, separately.

	NEG_nu_ M ± SD	NEG_po_ M ± SD	NEU_ne_ M ± SD	NEU_po_ M ± SD	POS_nu_ M ± SD	POS_ne_ M ± SD
YOUNG	0.934 ± 0.04	0.871 ± 0.06	0.926 ± 0.04	0.913 ± 0.04	0.964 ± 0.04	0.971 ± 0.04
OLD	0.787 ± 0.20	0.718 ± 0.21	0.805 ± 0.20	0.803 ± 0.21	0.808 ± 0.21	0.857 ± 0.19

## Data Availability

Materials and data will be publicly shared on request. The study was not pre-registered.
